# A Novel Multifunctional 5,6-Dimethoxy-Indanone-Chalcone-Carbamate Hybrids Alleviates Cognitive Decline in Alzheimer’s Disease by Dual Inhibition of Acetylcholinesterase and Inflammation

**DOI:** 10.3389/fnagi.2022.922650

**Published:** 2022-07-04

**Authors:** Chan Liu, Zhipei Sang, Hong Pan, Qin Wu, Yu Qiu, Jingshan Shi

**Affiliations:** ^1^Department of Pharmacology and Chemical Biology, Shanghai Universities Collaborative Innovation Center for Translational Medicine, Shanghai Jiao Tong University School of Medicine, Shanghai, China; ^2^Key Laboratory of Basic Pharmacology of Ministry of Education, Joint International Research Laboratory of Ethnomedicine of Ministry of Education, Zunyi Medical University, Zunyi, China; ^3^Department of Medicinal Chemistry, School of Pharmaceutical Sciences, Hainan University, Haikou, China

**Keywords:** Alzheimer’s disease, multi-target-directed ligands, acetylcholinesterase, amyloid-β, neuroinflammation

## Abstract

**Backgrounds:**

Alzheimer’s disease (AD) is a multifactorial neurodegenerative disease. The treatment of AD through multiple pathological targets may generate therapeutic efficacy better. The multifunctional molecules that simultaneously hit several pathological targets have been of great interest in the intervention of AD.

**Methods:**

Here, we combined the chalcone scaffold with carbamate moiety and 5,6-dimethoxy-indanone moiety to generate a novel multi-target-directed ligand (MTDL) molecule *(E)*-3-((5,6-dimethoxy-1-oxo-1,3-dihydro-2H-inden-2-ylidene)-methyl)phenylethyl(methyl) carbamate (named AP5). *In silico* approaches were used to virtually predict the binding interaction of AP5 with AChE, the drug-likeness, and BBB penetrance, and later validated by evaluation of pharmacokinetics (PK) *in vivo* by LC-MS/MS. Moreover, studies were conducted to examine the potential of AP5 for inhibiting AChE and AChE-induced amyloid-β (Aβ) aggregation, attenuating neuroinflammation, and providing neuroprotection in the APP/PS1 model of AD.

**Results:**

We found that AP5 can simultaneously bind to the peripheral and catalytic sites of AChE by molecular docking. AP5 exhibited desirable pharmacokinetic (PK) characteristics including oral bioavailability (67.2%), >10% brain penetrance, and favorable drug-likeness. AP5 inhibited AChE activity and AChE-induced Aβ aggregation *in vivo* and *in vitro*. Further, AP5 lowered Aβ plaque deposition and insoluble Aβ levels in APP/PS1 mice. Moreover, AP5 exerted anti-inflammatory responses by switching microglia to a disease-associated microglia (DAM) phenotype and preventing A1 astrocytes formation. The phagocytic activity of microglial cells to Aβ was recovered upon AP5 treatment. Importantly, chronic AP5 treatment significantly prevented neuronal and synaptic damage and memory deficits in AD mice.

**Conclusion:**

Together, our work demonstrated that AP5 inhibited the AChE activity, decreased Aβ plaque deposition by interfering Aβ aggregation and promoting microglial Aβ phagocytosis, and suppressed inflammation, thereby rescuing neuronal and synaptic damage and relieving cognitive decline. Thus, AP5 can be a new promising candidate for the treatment of AD.

## Background

Alzheimer’s disease (AD) is the primary cause of dementia that is characterized by progressive memory decline and cognitive dysfunction, often manifested pathologically by the deposition of extracellular amyloid-β (Aβ) plaques, the formation of intraneuronal neurofibrillary tangles, and neuroinflammation (Long and Holtzman, 2019; Guo et al., 2020). With increasing incidence as the global population ages, AD has imposed an enormous burden on health care systems (Collaborators, 2019). Although progress has been made on the mechanism of AD pathology, effective treatment is still limited (Livingston et al., 2020).

Alzheimer’s disease is a progressive multifactorial neurodegenerative disease in which multiple causative factors lead to cognitive decline and disease deterioration (Young et al., 2018; Savelieff et al., 2019). Therefore, targeting any single factor (e.g., Aβ, tau, neuroinflammation, etc.), even if successful, is not efficacious to reverse the disease escalation. Therefore, an effective cure may require a multipronged approach to combat multiple detrimental features in parallel. The multi-target-directed ligands (MTDLs), designed to modulate several molecule targets in parallel with a hybrid molecule, may be effective at lower doses due to additive or synergistic effects (Guzior et al., 2015). Several MTDLs for AD have been developed and are currently in clinical trials, including ladostigil tartrate (Schneider et al., 2019), eltoprazine, bexarotene, dextromethorphan, etc.

To reach the desired dual or multiple effects, the molecule targets of the MTDL drugs need to be charily selected. Acetylcholinesterase inhibitors (AChEIs) are usually taken into consideration for their symptomatic ameliorations (Pérez et al., 2015). AChE is a pivotal enzyme in the hydrolysis of the neurotransmitter acetylcholine (ACh). It has been shown that AChE possesses two active binding sites, including the peripheral anionic site (PAS), at the edge of the gorge and the catalytic anionic site (CAS) at the bottom (Johnson and Moore, 2006). AChE catalyzes the hydrolysis of ACh through its CAS. Accumulating pieces of evidence have demonstrated that PAS of AChE, responsible for non-cholinergic functions, greatly accelerates the assembly of Aβ into fibrils and promotes Aβ deposition (Inestrosa et al., 1996; Castro and Martinez, 2001; Galdeano et al., 2010). Blockade of PAS is effective for the prevention of Aβ plaque deposition by inhibiting Aβ assembling and consequently promoting Aβ clearance (Castro and Martinez, 2001). AChEIs aiming at both the PAS and CAS opened the prelude of AChEI-based MTDLs (Galdeano et al., 2010). It is well known that the carbamate fragment in rivastigmine is the inhibitory pharmacophore, which could covalently interact with CAS (Chaudhaery et al., 2010). In addition, the 5,6-dimethoxy-indanone moiety of donepezil was directed toward PAS of AChE (Mezeiova et al., 2019).

Neuroinflammation is not merely a consequence of disease progression but it also exacerbates the pathology of AD and accelerates cognitive impairment (Leng and Edison, 2021). Inflammation is linked with increases in Aβ generation, aggregation, and tau phosphorylation. Co-inhibition of inflammation and AChE has shown potential as a preventive treatment (Li et al., 2009). Chalcone (α-phenyl-β-benzoylethylene) is a simple molecule of many naturally existing compounds, such as flavonoids and isoflavonoids. Chalcone and its derivatives possess a diversity of pharmacological activities, including antioxidant activity, anti-inflammatory, and neuroprotective properties (Zhuang et al., 2017). Therefore, we designed a novel MTDL molecule *(E)*-3-((5,6-dimethoxy-1-oxo-1,3 -dihydro-2H-inden-2-ylidene)-methyl) phenylethyl (methyl) carbamate (thereafter named AP5) to fuse a carbamate moiety as CAS binding unit and a dimethoxy-indanone moiety as PAS binding unit with chalcone scaffold, hoping the molecule possess AChE inhibitory effect, anti-Aβ aggregation, and anti-inflammatory properties. The objective of this investigation is to determine whether ACh hydrolysis, Aβ deposition, and neuroinflammation can be resolved simultaneously by this MTDL and to demonstrate the effect of this novel MTDL on cognitive deficits in AD model mice.

## Materials and Methods

### Mouse

The APP/PS1 (APPswe/PSEN1dE9) double-transgenic mice of ages 6 months and age-, gender-matched C57BL/6 J as wild-type (WT) controls were bred at the Jiangsu ALF Biotechnology Co. (Nanjing, China). The C57BL/6J mice of ages 6 months for pharmacokinetics analysis were obtained from the Hunan SJA laboratory animal Co. (Changsha, China). Between 6 and 15 months of age, APP/PS1 mice exhibit a gender-based disparity in Aβ burden. Females develop a 5-fold (Aβ42) and 10-fold (Aβ40) increase in Aβ deposits in the cerebellum by 15 months as compared to males^1^. The sex divergence was also observed in other neuronal, survival, and autophagic markers. Thus, only male animals were used to avoid the influence of gender and minimize variability. All mice were fed with food and water available *ad libitum* on a 12-h light/dark cycle.

### The Multi-Target-Directed Ligand Drug

The novel MTDL molecule *(E)*-3-((5,6-dimethoxy-1-oxo-1,3-dihydro-2H-inden-2-ylidene) -methyl) phenylethyl (methyl) carbamate (AP5) was synthesized at the Department of Medicinal Chemistry (Hainan University, Haikou, China) using the methods reported in the patent cooperation treaty. The chemical structure of AP5 was validated by nuclear magnetic resonance at 400 MHz ^1^H NMR and 101 MHz ^13^C NMR (Supplementary Figure 1). ^1^H NMR (400 MHz, Chloroform-*d*) δ 7.59 (d, *J* = 2.1 Hz, 1H), 7.52-7.41 (m, 3H), 7.36 (s, 1H), 7.17 (d, *J* = 7.6 Hz, 1H), 7.02 (s, 1H), 4.04-3.95 (m, 8H), 3.57-3.42 (m, 2H), 3.13 (s, 1H), 3.05 (s, 1H), 1.27 (dt, *J* = 23.0, 6.9 Hz, 3H). ^13^C NMR (101 MHz, CDCl_3_) δ 193.03, 155.51, 151.86, 149.68, 144.99, 136.90, 136.13, 131.64, 131.03, 129.61, 127.57, 123.22, 122.79, 107.27, 105.12, 56.33, 56.20, 44.18, 34.35, 33.91, 32.02, 13.30, 12.51.

### Molecular Docking Assay

The computational study was performed to explore possible binding mechanisms of AChE for compound AP5 using the docking program. The key amino acids of PAS and CAS binding site of AChEinclude Tyr337, Tyr 341, His447, Gly 342, Trp 286, Glu 292, Phe295, Gly 122, and Ser 203 of the CAS binding site and Val 132, Tyr 124, Thr 83, Trp 86, Asp74, and Glu 202 of the PAS binding site (Mondal et al., 2018). Autodock Vina version 4.2 (Scripps research institute, Jupiter, FL, United States) was employed to carry out the molecule docking between AChE (PDB ID 2WHQ) and AP5. The Autodock Vina is an easy-to-use docking software and a graphical environment that mines the pharmacological interactions of potent molecules. Affinity grids of docking wrapped the whole AChE protein, and 200 conforms were searched. The conformation with the lowest binding energy is chosen as the optimal conformation. All pictures were observed and produced using PyMOL (DeLano Scientific, San Carlos, CA) and Discovery Studio 2019 (Biova, Waltham, MA, United States).

### Pharmacokinetics Analysis in Mice

To determine the pharmacokinetics of AP5 in mice, the mice were administered a single dose of AP5 by i.v. and oral gavage dose routes. The plasma and brain homogenate were detected by UPLC-TSQ/MS(Thermo Fisher Scientific, Waltham, MA, United States). The plasma drug concentrations were analyzed for PK data by DAS (Drug and Statistics) Version 3.0 (BioGuider Corporation, Shanghai, China). Bioavailability (F%) after oral administration was calculated as AUC_INF_ *_*p.o.*/_*AUC_INF i.v._**/**4 × 100. AUC_INF_ *_*p.o.*_* and AUC_INF i.v._ represent the area under the plasma concentration–time curve extrapolated to infinity after single-dose *per os* and intravenous injection, respectively. The extent of brain penetration was measured by the brain-to-plasma distribution ratio (B/P). The B/P value was calculated as: AUC_INFbrain/_AUC_INF plasma_ × 100. AUC_INF brain_ represents the area under the brain concentration–time curve extrapolated to infinity after a single-dose *per os*.

### Drug Treatment

APP/PS1 mice of ages 6 months were randomly divided into vehicle (APP/PS1, *n* = 10) and treatment groups (AP5, *n* = 10), which were intragastrically administered with AP5 (40 mg/kg) once daily for consecutive 5 months. AP5 was dissolved in distilled water containing 1% sodium carboxymethyl cellulose.

### Estimation of Acetylcholinesterase and Acetylcholine

The brain samples were weighed, homogenized in PBS solution, and centrifuged for 20 min at 25,000 *g* at 4°C. Amplex^®^ Red acetylcholinesterase/acetylcholine assay kit (A12217, Thermo Fisher Scientific) was used to detect AChE activity and ACh levels in the supernatant in accordance with the manufacturer’s instructions.

### Amyloid-β Aggregation and Disaggregation Assay

The effects of AP5 on Aβ fibril formation and pre-formed Aβ fibrils disaggregation were determined using a previously described method of Thioflavin T (ThT, T3516, Sigma Aldrich, St. Louis, MO, United States) fluorometry (Jan et al., 2010).

### Dot Blot

The formation of Aβ40 or Aβ42 oligomers was monitored by dot blot with oligomer-specific antibody. Briefly, the resulting Aβ film was dissolved in ddH_2_O to form a 100 μM solution, and then the oligomerization reaction (Kayed et al., 2003) was initiated in absence or presence of different concentrations of AP5. An aliquot (2 μL) of each solution was dripped on a nitrocellulose membrane. The membrane was blocked with 5% non-fat milk in TBS for 1 h, incubated with the anti-β amyloid (MOAB-2, NBP2-13075SS, Novus Biologicals, Colorado, United States), anti-amyloid oligomers (A11, NBP1-97930, Novus Biologicals), or anti-amyloid fibrils (OC, NBP1-97930, Novus Biologicals) antibodies overnight at 4°C. The membrane was then incubated using HRP-conjugated secondary antibodies at room temperature for 1 h and then developed with ECL.

### Behavioral Tests

To investigate whether AP5 treatment plays a key role in cognition improvement of APP/PS1 mice models, the five different behavioral tests were sequentially carried out in the following order: open field, novel object recognition task, spatial object-location task, Y-maze, and Morris water maze (MWM). AP5 was also administered daily during the test periods. A set of video analysis systems (TopScan, CleverSys, United States) was used throughout the behavioral tests.

### Preparation of Brain Samples

After behavioral tests, all animals were anesthetized with sodium pentobarbital (50 mg/kg intraperitoneally) and decapitated. The left hemisphere was fixed in 4% paraformaldehyde for 48 h. The right hemispheric hippocampus and cortex were collected separately, snap-frozen in liquid nitrogen, and then stored at −80°C until the analyses of protein and gene expression.

### Immunohistochemistry and Immunofluorescence

The fixed hemibrains were paraffin-embedded and sectioned in the coronal plane. The slices were deparaffinized with xylene and rehydrated by a series of gradient alcohols. Antigen retrieval through microwave boiling was performed with 10 mM sodium citrate solution at pH 6.0. The slices were cooled down to room temperature and then rinsed in PBS solution. Then the slices were incubated in 10% goat serum blocking solution for 1 h followed by primary antibodies overnight at 4°C. The next day, the slides were labeled with biotinylated or fluorescent secondary antibodies. Slices were washed three times with PBS before being mounted. Images were captured using a SpinSR10 scanner (Olympus, Tokyo, Japan).

### Thioflavin S Staining

The hemisphere slices were stained with 0.5% fluorescent amyloid dye Thioflavin S (ThS, T1892, Sigma Aldrich) in the dark for 10 min followed by three washes with 50% ethyl alcohol and a final wash in PBS. Image analysis of the number and size of cortical and hippocampal ThS positive plaques was carried out on Image J FIJI software with “analyze particles” function.

### Enzyme-Linked Immunosorbent Assay

For detecting the pathological soluble or insoluble Aβ in mice, sequential extractions were performed as follows: the half of right hippocampus and cortex from three groups were homogenized in TBS solution containing 5 mM EDTA, phosphatase inhibitor (P1050, Beyotime, Shanghai, China), and protease inhibitor cocktail (P1010, Beyotime) followed by centrifugation at 25,000*g* at 4°C for 1 h. The supernatant was collected as a TBS fraction and the resulting pellet was solubilized in 70% formic acid (FA), sonicated until the lysis buffer was clear, followed by centrifugation at 25,000g at 4°C for 1 h. The supernatant was collected as the FA fraction and neutralized (1:20) in a neutralization buffer (1 M tris base, 0.5 M Na_2_HPO_4_, 0.05% NaN_3_). Human Aβ40 and Aβ42 were determined by ELISA kits (E-EL-H0452c and E-EL-H0453c, Elabscience, Wuhan, China) in accordance with the manufacturer’s instructions. In addition, the levels of IL-1β (88-7013-88, Invitrogen), IL-6 (EK206HS-96, Multi sciences, HangZhou, China), and TNF-α (88-7324-88, Invitrogen) in TBS fraction were also determined by ELISA.

### Western Blotting

Frozen hippocampus and cortex were homogenized in pre-cooling RIPA buffer (P0013B, Beyotime) supplemented with 1 mM phenylmethanesulfonyl fluoride (PMSF) and cocktail of protease and phosphatase inhibitors. Protein samples were separated in 10% or 12% SDS-PAGE and then transferred to PVDF membranes. The membranes were incubated with 5% fat-free milk blocking solution for 1 h at room temperature and then probed with primary antibodies overnight at 4°C. The membranes were incubated with the corresponding HRP-conjugated secondary antibodies at room temperature for 1 h and developed with ECL. Densitometric analysis was performed using Quantity One software (Bio-Rad, Berkeley, CA, United States).

### RNA-Sequence

Hippocampal specimens from WT, APP/PS1, and AP5 mice stored in dry ice were sent to Beijing Genomics Institute (Wuhan, China), and the total RNA was extracted for RNA sequencing on BGISEQ-500 system. Moreover, the differentially expressed genes between APP/PS1 and AP5 were analyzed after RNA sequencing.

### Statistical Analysis

Prism 8.3.0 Software (GraphPad Software Inc., La Jolla, CA, United States) was used for statistical analyses. Data are expressed as mean ± standard error of the mean (SEM). The statistical difference between the two independent groups was analyzed with the two-tailed unpaired *t*-test. Samples among more than two groups were compared using parametric one- or two-way ANOVA followed by the Turkey’s *post hoc* test. *Post hoc* tests were conducted when the F value achieved the necessary level (*p* < 0.05) and there was no significant variance inhomogeneity. *p* Values less than 0.05 were considered statistically significant.

## Results

### Design, Drug-Likeness, and Pharmacokinetic Evaluation of 5,6-Dimethoxy-indanone-chalcone-carbamate Hybrids as a Novel Multi-Target-Directed Ligand

**FIGURE 1:**
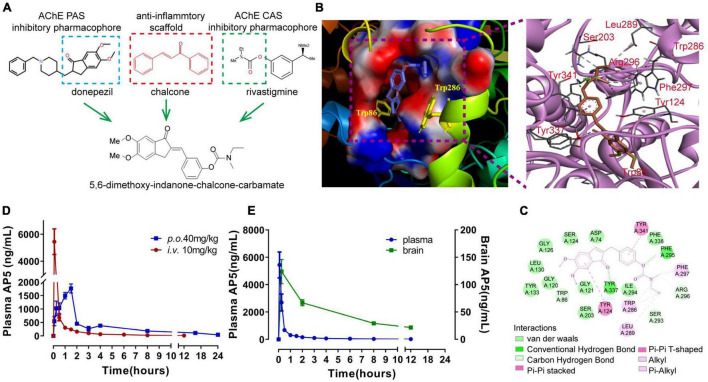
Design, molecular docking, and pharmacokinetic evaluation of *(E)*-3-((5,6-dimethoxy-1-oxo-1,3-dihydro-2H-inden-2-ylidene)-methyl)phenylethyl(methyl) carbamate (named AP5). **(A)** Design strategy for AP5. **(B)** 3D docking diagram of AP5 and AChE active gorge (left), and the main amino acids involved in the interaction with AP5 (right). **(C)** 2D docking diagram of AP5 and AChE. **(D)** Line graphs showing changes in plasma concentration of AP5 after single-dose intravenous injection (i.v., 10 mg/kg) or *per os* (*p.o.*, 40 mg/kg). Bioavailability (F%) after oral administration was calculated as AUC_INF_ *_*p.o.*_***/**AUC_INF i.v._**/**4 × 100%. AUC_INF_ *_*p.o.*_* and AUC_INF i.v._ represent area under the plasma concentration-time curve extrapolated to infinity after single-dose *per os* and intravenous injection, respectively. **(E)** Line graphs showing changes in concentrations of AP5 in plasma and brain after single-dose oral administration (40 mg/kg). The extent of brain penetration was measured by brain-to-plasma distribution ratio (B/P). The B/P value was calculated as AUC_INFbrain_**/**AUC_INF plasma_ × 100%. AUC_INF brain_ represents the area under the brain concentration-time curve extrapolated to infinity after a single-dose *per os*. *n* = 4 mice per time point.

We designed and generated a new MTDL molecule *(E)*-3-((5,6-dimethoxy-1-oxo-1,3-dihydro-2H-inden-2-ylidene)-methyl)phenylethyl(methyl)carbamate (named AP5) by binding 5,6-dimethoxy-indanone and carbamate moiety with chalcone molecular scaffolds (Figure 1A). Then, we first explored the binding potential of AP5 with the active pocket of AChE (PDB ID 2WHQ) through molecular docking. The molecular docking result showed that AP5 had a moderate binding affinity with the AChE binding pocket (Figure 1B) with the lowest binding energy of −10.64 kcal/mol compared to donepezil (−11.94kcal/mol) and rivastigmine (−8.55kcal/mol). The carbamate moiety of AP5 was buried deep into the catalytic site of AChE; however, 5,6-dimethoxy -indanone moiety contacted the peripheral sites at the edge of the gorge (Figure 1B). Based on active amino acids in the vicinity of CAS and PAS of AChE (Mondal et al., 2018), the carbamate moiety of AP5 bound with Phe295 and Arg296 of CAS *via* the hydrogen bond interaction, with 5,6-dimethoxy-indanone moiety of AP5 binding to Tyr337 and Trp86 of PAS (Figure 1C). The other hydrophobic interaction was observed between 5,6-dimethoxy-indanone and side-chain Tyr124 located in the PAS binding region. The carbamate group contacted the Trp286 and Leu289 in the CAS region by hydrophobic interactions. AP5 also exhibited van der Waals interactions with Asp74, Gly120, Ser125 of PAS, and Ile294, Phe338 of CAS. Taken together, the docking analysis suggests that AP5 fits well in the active pocket of AChE by interacting with the peripheral and catalytic site residues simultaneously, which indicates its dual inhibition and high potency.

Combining several pharmacophores may lead to a large molecule, which may compromise drug-likeness properties (Benek et al., 2020). Drug-likeness assessment *in silico* by Lipinski’s rule of five (Lipinski et al., 2001) showed that AP5 complied with Lipinski’s rule of five (Supplementary Table 1). Moreover, *in silico* assessment of blood–brain barrier (BBB) penetration showed that BBB-score >0.02 (Gupta et al., 2019) (Supplementary Table 1). These data indicate that AP5 holds appropriate drug-likeness properties and BBB permeability.

We next determine the pharmacokinetic profile of AP5 in male C57BL/6J mice given a single intravenous (i.v.) dose of 10 mg/kg or single *per os* (*p.o.*) dose of 40 mg/kg, respectively. After a single oral dose, AP5 concentration in the plasma reached the peak (2088 ng/mL) at 1.5 h by LC-MS/MS analysis. Importantly, we determined AP5 in the brain homogenate with the highest concentration of 95.3 ng/mL at 2 h (Figures 1D,E). PK parameters revealed that oral AP5 holds high systemic clearance (Cl = 2.756 L/hr/kg), moderate volume of distribution (Vss = 18.79 L/kg), favorable oral bioavailability F = 67.2%, and adequate oral brain penetration (B/P = 10.23%) (Figures 1D,E and Supplementary Table 2). Taken together, AP5 showed adequate BBB permeability, suitable PK properties, and drug-likeness properties, which supports the potential to develop AP5 as a promising candidate for studies in the AD model.

### AP5 Inhibits Acetylcholinesterase Activity and Blocks Aβ Oligomerization or Fibrillization

**FIGURE 2:**
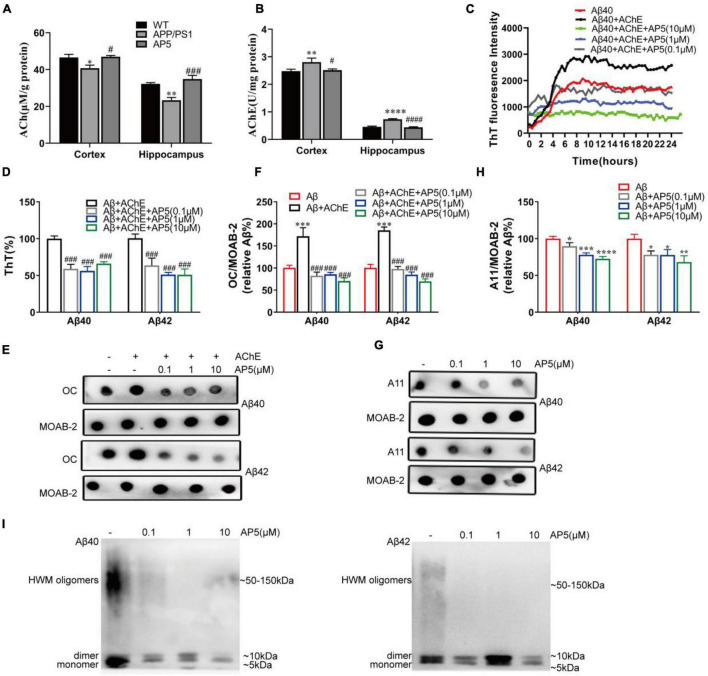
AP5 inhibits AChE activity and blocks AChE-induced Aβ fibrillization and self-oligomerization. **(A)** The AChE activity and **(B)** ACh levels of the cortex and hippocampus from WT, APP/PS1, and AP5 mice. *P < 0.05, **P < 0.01, ****P < 0.0001 compared with WT mice, ^#^*P* < 0.05, ^###^*P* < 0.001, ^####^*P* < 0.0001 compared with APP/PS1 mice by one-way ANOVA followed by a Turkey’s *post hoc* test, *n* = 6 mice per group. **(C)** The ThT assessment of AChE-induced Aβ40 fibril formation kinetics during 24 h. Triple replicates per group at each time point. **(D)** The disaggregation assessment on pre-formed Aβ40- and Aβ42-AChE fibrils complex using ThT binding. Triple replicates per group. **(E)** Dot blot images and **(F)** quantification of pre-formed Aβ40-and Aβ42-AChE fibrils complex with or without AP5 using an amyloid fibrils-specific antibody (OC). Triple replicates per group. **(G)** Dot blot images and **(H)** quantification of Aβ40 and Aβ42 oligomers with or without AP5 using an oligomer-specific antibody (A11). *P < 0.05, **P < 0.01, ***P < 0.001 compared with Aβ alone, ^###^*P* < 0.001 compared with Aβ + AChE group by one-way ANOVA followed by a Turkey’s *post hoc* test, *triple* replicates per group. **(i)** Immunoblot analysis of Aβ40 (left)and Aβ42 (right) oligomers with or without AP5 using an anti-amyloid-β antibody (MOAB-2). Triple replicates per group. HMW, high molecular weight.

As result of carbamate pharmacophore of AP5 binding to CAS of AChE, we further examined the effects of AP5 on AChE activity in APP/PS1 mice. Consistent with previous study (Shi et al., 2018), our data showed that AChE activity in the cortex and hippocampus was markedly increased in APP/PS1 mice (Figure 2A), which led to a reduction in the ACh level compared to WT mice (Figure 2B). AP5-treated mice exhibited decreased activity of AChE compared with APP/PS1 mice in the cortex (F_2,15_ = 7.730 *p* = 0.0049) and hippocampus (F_2,15_ = 29.21, *p* < 0.0001), and elevated levels of ACh in cortex (F_2,15_ = 7.886, *p* = 0.0046) and hippocampus (F_2,15_ = 22.51, *p* < 0.0001; Figures 2A,B).

It is reported that Aβ could bind to AChE through PAS and induces its fibrillization (Inestrosa et al., 1996; Johnson and Moore, 2006). Propidium and fasciculin, PAS inhibitors, are capable of preventing the effect of AChE on Aβ fibril aggregation process (Inestrosa et al., 1996). As 5,6-dimethoxy-indanone moiety of AP5 binds AChE at PAS, we next evaluated whether AP5 exerted an inhibitory effect on Aβ fibrillogenesis induced by AChE. Firstly, we analyzed the inhibition of AP5 on Aβ fibril formation induced by AChE using 24-h kinetic ThT fluorescence assay. As shown in Figure 2C, the incubation of Aβ with AChE resulted in an increase in fluorescence intensity, confirming that AChE was able to promote Aβ assembly into the fibrils. Conversely, co-incubation of AP5 and Aβ40 with AChE substantially lowered the ThT intensity in a dose-dependent manner, indicating that AP5 inhibited the enhancement of Aβ fibrillization triggered by AChE. Besides, the effects of AP5 on disaggregation of pre-formed Aβ40 or Aβ42 fibrils were determined. Aβ40 or Aβ42 was firstly pre-aggregated with AChE for 2 days before co-incubation with AP5. In the presence of AP5, Aβ fibrils were disaggregated as shown by a marked decline of ThT fluorescence intensity in Aβ40 (F_3,8_ = 49.64, *p* < 0.0001) and Aβ42 (F_3,12_ = 43.32, *p* < 0.0001; Figure 2D). This observation was further confirmed by the dot blot assay with the amyloid fibrils-specific antibody (Aβ40, F_4,10_ = 93.92, *p* < 0.0001) (Aβ42, F_4,10_ = 161.9, *p* < 0.0001; Figures 2E,F).

To study whether AP5 could inhibit Aβ self-oligomerization, the dot blot assay was carried out with the amyloid oligomer-specific antibody. Monomeric Aβ40 or Aβ42 was incubated with or without AP5 under conditions that led to oligomerization. As indicated in Figures 2G,H, AP5 decreased the levels of oligomeric Aβ40 (F_3,8_ = 34.91, *p* < 0.0001) and Aβ42 (F_3,8_ = 13.92, *p* = 0.0015).This result was further corroborated by immunoblot detection that discriminated between Aβ oligomers and monomers. The synthetic oligomer preparations comprised a mixture of low molecular weight oligomers and high molecular weight (HMW) oligomers. HMW Aβ oligomers were observed as a smear on SDS-PAGE, typically ranging in molecular weight from 50 to 150 kDa (Ferreira et al., 2015). As shown in Figure 2I, a 5-kDa Aβ monomer band, a 10-kDa Aβ dimer band, and a smear were detected. In the presence of AP5, the intensity of the Aβ dimers and smear were decreased, demonstrating that AP5 specifically blocked Aβ oligomerization. Taken together, these data suggested that AP5 might not only elevate the Ach level but also interfered with toxic Aβ aggregation.

### AP5 Reduces Aβ Deposition Without Altering APP Processing in APP/PS1 Mice

**FIGURE 3:**
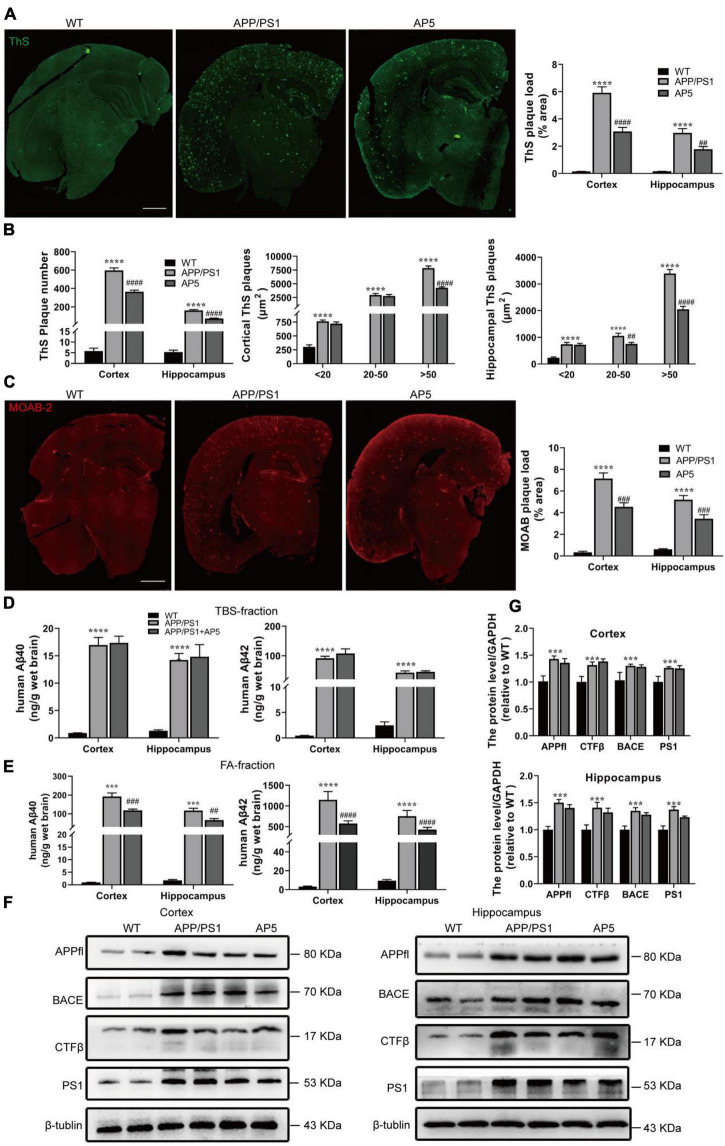
AP5 alleviates Aβ deposition without influencing APP processing. **(A)** Representative images and quantification of dense-core amyloid plaque by Thioflavin S (ThS) staining. Scale bar: 500 μm. **(B)** Quantification of the number and size of ThS-positive plaques. **(C)** Representative images and quantification of Aβ plaque immunofluorescence using MOAB-2 antibody. Scale bar: 500 μm. **(D)** The amount of Aβ40 and Aβ42 in TBS-fractions extracted from the cortex and hippocampus was examined by ELISA analyses. **(E)** The amount of Aβ40 and Aβ42 in FA-fractions extracted from the cortex and hippocampus was examined by ELISA analyses. **(F)** Protein expression of APPfl, CTFβ, BACE, and PS1 in the cortex (left) and hippocampus (right) were examined by western blot. **(G)** Protein expression of APPfl, CTFβ, BACE, and PS1 in the cortex (up) and hippocampus (down)were quantified with Quantity One software. ***P < 0.001, ****P < 0.0001 compared with WT mice, ^##^*P* < 0.01, ^###^*P* < 0.001, ^####^*P* < 0.0001 compared with APP/PS1 mice by one-way ANOVA followed by a Turkey’s *post hoc* test, *n* = 6 mice per group.

We next evaluated the overall effect of AP5 on Aβ pathology. Brain slices were stained with Thioflavin S (ThS) to detect dense-core fibrillar amyloid plaques. Indeed, we observed there was a pronounced reduction in the load (cortex, F_2,5_ = 86.47, *p* < 0.0001; hippocampus, F_2,15_ = 44.26, *p* < 0.0001) and number (cortex, F_2,15_ = 246.6, *p* < 0.0001; hippocampus, F_2,15_ = 150.8, *p* < 0.0001) of ThS-positive plaque in APP/PS1mice treated with AP5 (Figures 3A,B). Further, this reduction was most pronounced for larger (> 50μm^2^ in area) plaques in the cortex (F_2,15_ = 253.2, *p* < 0.0001) and hippocampus (F_2,15_ = 251.6, *p* < 0.0001). Similarly, immunofluorescent staining of Aβ peptide by MOAB-2 antibody, which recognizes the unaggregated, oligomeric, and fibrillar form of Aβ, indicated that the area occupied by unaggregated and aggregated Aβ was also decreased in cortex (F_2,15_ = 82.42, *p* < 0.0001) and hippocampus (F_2,15_ = 64.06, *p* < 0.0001) of the AP5-administered AD mice(Figure 3C).

To further detect Aβ aggregation in these mice, we performed a serial extraction of cortex and hippocampus. TBS fraction contains more soluble Aβ species while the 70% FA fraction contains insoluble Aβ aggregates. APP/PS1 mice had significantly higher Aβ40 and Aβ42 levels in TBS- and FA-fractions (Figures 3D,E). Furthermore, the majority of Aβ in APP/PS1 mice was fractioned in the FA-soluble fractions (Figure 3E), indicating its deposition in the amyloid plaque. In addition, as shown in Figure 3E, the concentrations of insoluble Aβ40 (cortex, F_2,15_ = 82.42, *p* < 0.0001; hippocampus, F_2,15_ = 46.30, *p* < 0.0001) and Aβ42 (cortex, F_2,15_ = 132.4, *p* < 0.0001; hippocampus, F_2,15_ = 112.2, *p* < 0.0001) were markedly decreased in FA-fractions, but no marked differences were determined in TBS-fraction after AP5 administration (Figure 3D).

Next, to clarify the mechanism that contributed to the reduction in Aβ following AP5 administration, we assessed APP processing by western blot. The results showed there were no significant changes in the expression levels of the amyloid precursor protein (APP full length, APPfl), CTFs (C-terminal fragments, CTFβ), β-site-APP cleaving enzyme (BACE), and catalytic subunit of the gamma-secretase complex (presenilin 1) between APP/PS1 and AP5-treated APP/PS1 mice (Figures 3F,G). These data suggest that AP5 did not alter APP expression or processing. Overall, these results demonstrated that AP5 administration mitigates Aβ load without influencing Aβ production.

### AP5 Alleviates Neuroinflammation of APP/PS1 Mice

On the basis of the structural properties of AP5, which contains anti-inflammatory and antioxidative chalcone structure, we examined the effects of AP5 on neuroinflammation in APP/PS1 mice. Firstly, the brain slices were immunostained with ionized calcium-binding adapter molecule 1 (Iba1) antibody and glial fibrillary acidic protein (GFAP) to investigate the effects of AP5 on microgliosis and astrogliosis, respectively. We morphologically classified Iba1^+^ microglia to quantify the microglia activation state and observed that APP/PS1 mice exhibited a significantly elevated proportion of intermediate, amoeboid, or round microglial cells, showing an increase in microglia activation. For the APP/PS1 mice treated with AP5, we found a marked decrease in the proportion of activated phenotype (F_2,15_ = 10.71, *p* = 0.0013; Figures 5A,B). Consistent with a previous study (Chun et al., 2020), quantitative analysis of individual reactive astrocyte morphology using Sholl analysis showed elevated ramification index (F_2,15_ = 44.12, *p* < 0.0001) and the sum of intersects (F_2,15_ = 82.97, *p* < 0.0001) in APP/PS1 mice. Similar to microglia, these reactive phenotypes of astrocytes were attenuated by AP5 treatment (Figures 5C,D). However, the ending radius, an indicator of astrocyte territory, was not changed in APP/PS1 mice.

Next, we determined the levels of several proinflammatory cytokines (TNF-α, IL-6, and IL-1β) implicated in AD. A greatly increased secretion of TNF-α (F_2,15_ = 107.0, *p* < 0.0001), IL-6 (F_2,15_ = 54.31, *p* < 0.0001), and IL-1β (F_2,15_ = 25.01, *p* < 0.0001) were detected in APP/PS1 mice, which were markedly reduced by AP5 administration (Figure 5E). Together, these results show AP5 attenuated neuroinflammatory responses in the AD mice model.

It has been reported that microglia clustered in the vicinity of Aβ plaque form a protective barrier that compacts amyloid fibrils and reduces their toxicity (Yuan et al., 2016). Thus, we quantified the Aβ plaque-associated microglia by immunofluorescent co-staining with MOAB-2 and Iba1. The increased clustering of microglia surrounding a plaque was observed in the brain of AP5-treated APP/PS1 mice (Figures 5F,G). Microglia clustering around plaques facilitates extracellular Aβ clearance (Zhong et al., 2019). Thus, we further investigated whether the increased microglia recruitment in the vicinity of plaques upon AP5 treatment can promote Aβ phagocytosis. As shown in Figures 5J,K, the area of CD68 (a marker of phagocytic activity of microglia) within the microglia was increased, suggesting that AP5 treatment enhances microglial response to Aβ plaques and phagocytic activity. These results revealed that AP5 decreased the microglial activation and enhanced the clustering of microglia around Aβ plaque, thereby diminishing plaque-associated neurotoxicity and promoting microglia phagocytosis and clearance of Aβ. Besides, we found there was no statistical difference in the number of astrocytes around Aβ plaque (Figures 5H,I).

### AP5 Rescues the Neuron and Synapse Damage in the APP/PS1 Mice

**FIGURE 4:**
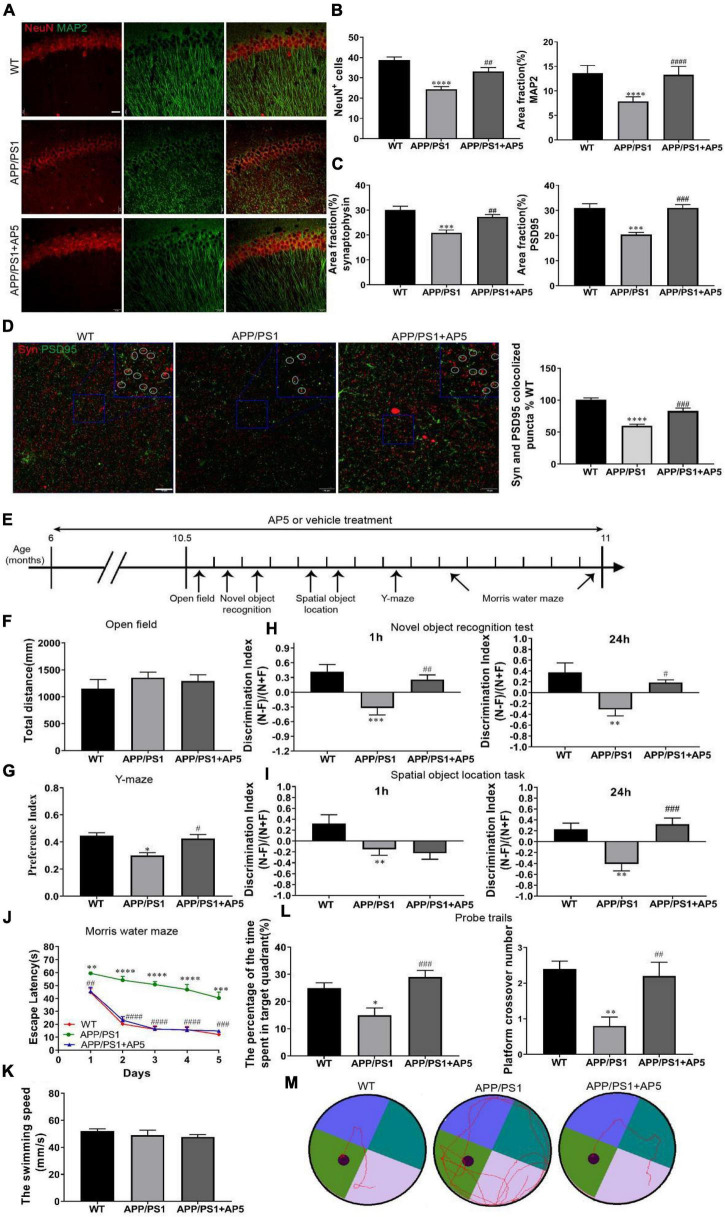
AP5 restores neuronal and synaptic loss and improves cognitive deficits in APP/PS1 mice. **(A)** Representative fluorescent photomicrographs of NeuN, neuronal marker, and MAP2, dendritic marker, co-immunostaining in the CA1 region of hippocampus. Scale bar: 20 μm. **(B)** Quantification of neuron survival by counting the average number of NeuN + cells and MAP2 fluorescence intensity in the CA1 pyramidal cell layer. **(C)** Representative fluorescent photomicrographs and quantification of synaptophysin (Syn) and PSD95 co-immunostaining in CA3 area of hippocampus. Scale bar: 10 μm. Co-localized puncta are marked by circles. **(D)** Quantitative analysis of the Syn and PSD95 fluorescence intensity. *P < 0.05, ***P < 0.001, ****P < 0.0001 compared with WT mice, ^#^*P* < 0.05, ^##^*P* < 0.01, ^###^*P* < 0.001 by one-way ANOVA followed by a Turkey’s *post hoc* test, *n* = 6 mice per group. **(E)** The timeline of the various behavioral experimental procedure. **(F)** Quantification of the locomotor activity in the open field test. **(G)** Quantification of the preference index at 3 h in the Y-maze. **(H)** Quantification of the discrimination index at 1 h and at 24 h in the novel object recognition test and **(I)** the spatial object location task. *P < 0.05, **P < 0.01 compared with WT mice, ^#^*P* < 0.05, ^###^*P* < 0.001 compared with APP/PS1 mice by one-way ANOVA followed by a Turkey’s *post hoc* test, *n* = 10 mice per group. **(J)** The escape latency of each group was detected in the Morris water maze (MWM) for 5 consecutive days. **P < 0.01, ***P < 0.001, ****P < 0.0001 compared with WT mice, ^##^*P* < 0.01, ^###^*P* < 0.001, ^####^*P* < 0.0001 compared with APP/PS1 mice by two-way ANOVA followed by a Turkey’s *post hoc* test, *n* = 10 mice per group. **(K)** Quantification of the swimming speed in the MWM training test. **(L)** The percentage of the time spent in the target quadrant(left) and platform crossover number (right) in the probe trials are shown. *P < 0.05, **P < 0.01 compared with WT mice, ^#^*P* < 0.05, ^##^*P* < 0.01, ^###^*P* < 0.001 compared with APP/PS1 mice by one-way ANOVA followed by a Turkey’s *post hoc* test, *n* = 10 mice per group. **(M)** Representative motion tracking of the probe trials.

Aβ aggregation in the brain is a key initiating step in the pathogenesis of AD, which induces many downstream detrimental events such as oxidative stress, neuroinflammation, synaptic impairment, neuronal degeneration, and eventual cognitive defects (Guo et al., 2020). Therefore, we next examined whether the protective effects of AP5 against Aβ-induced neurotoxicity was reflected at the neuronal and synaptic level. Firstly, to determine the effects of AP5 on neuron loss and degeneration in APP/PS1 mice, we carried out co-immunostaining with NeuN and MAP2. Remarkably, we detected that the average number of NeuN^+^ cells was reduced (F_2,15_ = 21.55, *p* < 0.0001), and MAP2 signals were also significantly disrupted (F_2,15_ = 30.17, *p* < 0.0001) in the hippocampal CA1 pyramidal layer of APP/PS1 mice, but these changes were restored in AP5-treated APP/PS1 mice (Figures 4A,B). Consistent with these results, the protein expression of NeuN and MAP2 was markedly increased in the hippocampus of AP5-treated mice (Supplementary Figures 2A,B).

**FIGURE 5:**
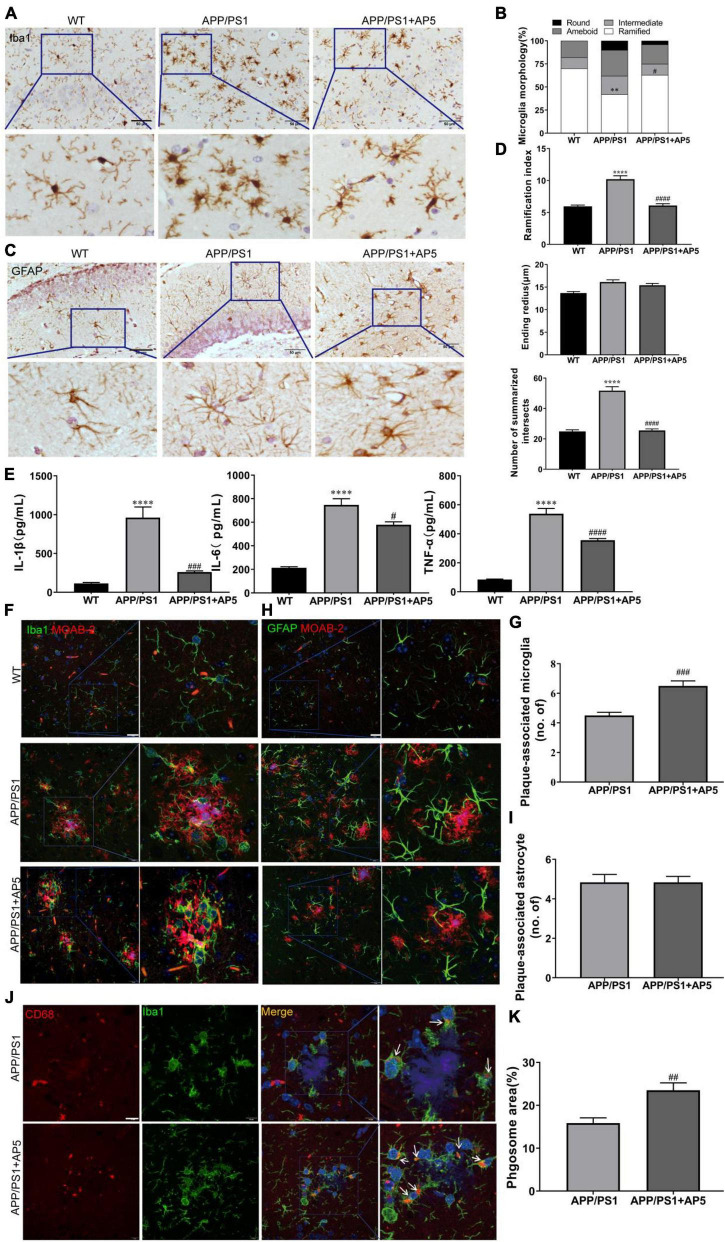
AP5 attenuates the neuroinflammation state of APP/PS1 mice. **(A)** Representative images of Iba1 immumohistochemistry staining from WT, APP/PS1, and AP5 mice. Scale bar: 50 μm. **(B)** Quantification of morphological classification in Iba1 positive microglial cells. **(C)** Representative images of GFAP immumohistochemistry staining from WT, APP/PS1, and AP5 mice. Scale bar: 50 μm. **(D)** Quantification of the summarized intersects, ramification index, and ending radius in GFAP positive astrocytes. **(E)** The levels of TNF-α, IL-6, and IL-1β. **P < 0.01, ****P < 0.0001 compared with WT mice, ^#^*P* < 0.05, ^###^*P* < 0.001, ^####^*P* < 0.0001 compared with APP/PS1 mice by one-way ANOVA followed by a Tukey’s *post hoc* test, *n* = 6 mice per group. **(F)** Representative images of MOAB-2 and Iba1 co-staining and **(G)** quantification of the number of plaque-associated microglia from APP/PS1 and AP5 mice. Scale bar: 20 μm. **(H)** Representative images of MOAB-2 and GFAP co-staining and **(i)** quantification of the number of plaque-associated astrocytes from APP/PS1 and AP5 mice. Scale bar: 20 μm. **(J)** Representative images of CD68 and Iba1 co-staining and **(K)** quantification of CD68 area within microglia from the APP/PS1 and AP5 mice. Scale bars: 20 μm. ^##^*P* < 0.01, ^###^*P* < 0.001 compared with APP/PS1 mice by unpaired t-test, *n* = 6 mice per group.

Next, we investigated the efficacy of AP5 against synaptic pathology in APP/PS1 mice. Synaptophysin and PSD95 are two membrane protein markers located in the presynaptic and postsynaptic cells, respectively. Co-immunofluorescence of synaptophysin with PSD95 revealed reduced pre- (F_2,15_ = 16.1, *p* = 0.002), post- (F_2,15_ = 20.04, *p* < 0.0001), and double-positive-synaptic puncta (F_2,15_ = 37.82, *p* < 0.0001) in APP/PS1 mice, and the synapse damage was markedly recovered by AP5 (Figures 4C,D). Western blot further indicated that the expression levels of synaptophysin and PSD95 in the hippocampus of AP5-treated mice were more than that in the APP/PS1 mice, respectively (Supplementary Figures 4A,B).

These results suggested that AP5 prevented or reversed neuronal and synaptic damage, which might play a pivotal role in ameliorating downstream cognitive defects.

### Chronic Dosing of AP5 From the Early Stages of β-Amyloidosis Alleviates Cognitive Deficits of APP/PS1 Mice

We next assessed whether the beneficial effects of AP5 in decreasing Aβ plaque deposition and neuroinflammation are accompanied by cognitive improvement in APP/PS1 mice (Figure 4E). Firstly, we evaluated the general locomotor activity of WT, APP/PS1, and AP5 mice through the open field test, and no difference in the total distance traveled (F_2,27_ = 0.6091, *p* = 0.5511) was observed among groups, indicating that AP5 administration did not elicit any influence on the general locomotor activity of the mice (Figure 4F). Furthermore, we employed Y-maze to evaluate spatial recognition memory. AP5-treated mice demonstrated a marked elevation of their preference index (F_2,27_ = 11.07, *p* = 0.0003) relative to APP/PS1 mice after training for 3 h, suggesting the improvement of short-term spatial recognition memory (Figure 4G).

Moreover, we used the novel object recognition task and the spatial object location task to evaluate recognition memory. APP/PS1 mice demonstrated impaired short- and long-term recognition memory by a significant decline of their discrimination index at 1 h and 24 h compared with WT mice (Figures 4H,I). AP5-treated APP/PS1 mice performed significantly better than APP/PS1 mice without treatment in the novel object recognition task (F_2,27_ = 8.215, *p* = 0.0016) and the spatial object location task (F_2,27_ = 11.05, *p* = 0.0003) at 24 h (Figures 4H,I). In addition, the discrimination index in AP5-treated AD mice for novel object recognition was significantly higher (F_2,27_ = 9.417, *p* = 0.0008) than APP/PS1 mice at 1 h (Figure 4H). These data indicate an improvement in short- and long-term recognition memory upon AP5 treatment.

We examined the reference memory through the Morris water maze (MWM). As indicated in Figure 4J, APP/PS1 mice exhibited marked spatial learning and memory decline by longer escape latency in the successive platform learning trials, less time spent in the target quadrant, and lower platform crossover number in the probe trial (Figures 4L,M). The swimming velocity during the successive training period was indistinguishable (F_2,27_ = 0.7216, *p* = 0.4951) between each group (Figure 4K). After 5 months of treatment with AP5, the mice performed much better in escape latency including the time effect (F_2.797,75.51_ = 66.46, *p* < 0.0001) and group effect (F_2,27_ = 79.06, *p* < 0.0001) by two-way ANOVA, platform crossing number (F_2,27_ = 8.695, *p* = 0.0012), and time spent in the target quadrant (F_2,27_ = 9.236, *p* = 0.0009) (Figure 4L), suggesting that AP5 slows cognitive decline in AD mice.

### AP5 Regulates Gene Transcription in APP/PS1 Mice

**FIGURE 6:**
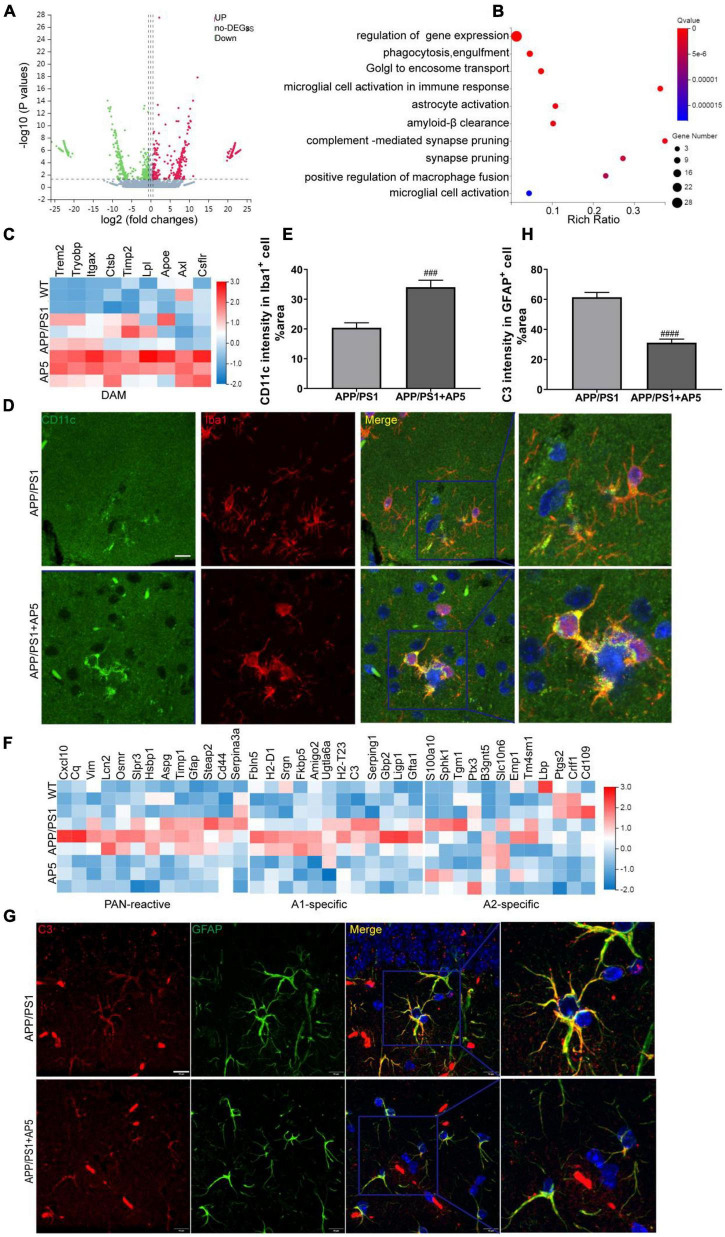
Transcriptomic profiling overview of hippocampi from WT, APP/PS1, and AP5 mice. **(A)** Volcano plots show the differentially expressed mRNAs in the hippocampus of AP5 vs. APP/PS mice. (Vertical lines represent ≥1.5-fold changes, and the horizontal line represents *p* < 0.05). **(B)** Gene ontology(GO) annotation of differentially expressed mRNAs in the hippocampus of AP51 vs. APP/PS mice. The top 10 GO terms are shown. **(C)** Hierarchical clustering heatmap of DAM genes from RNA-seq in hippocampus among WT, APP/PS1, and AP5 mice. **(D)** Representative images of CD11c, a DAM marker, and Iba1 co-immunostaining and **(E)** quantification of CD11c area within microglia from APP/PS1 and AP5 mice. Cell nuclei are shown in blue (DAPI). Scale bar:10 μm. **(F)** Hierarchical clustering heatmap of reactive astrocyte genes from RNA-seq in hippocampus among WT, APP/PS1, and AP5 mice. **(G)** Representative images of C3, A1 astrocyte marker, and GFAP co-immunostaining and **(H)** quantification of C3 area within astrocyte from APP/PS1 and AP5 mice. Scale bar:10 μm.^###^*P* < 0.001,^####^*P* < 0.001 compared with APP/PS1 mice by unpaired *t*-test, *n* = 6 mice per group.

Given the multitargeted effects of AP5 that we observed, we next explored the underlying molecular mechanism by high-throughput bulk RNA sequencing (RNA-seq) of the hippocampal tissues. A total of 17,981 genes were identified in the hippocampi from above WT, APP/PS1, and AP5 mice. Volcano plots depicted that 933 genes were differentially expressed, of which 425 genes were induced and 508 genes were suppressed in APP/PS1 mice treated with AP5 treatment compared with APP/PS1 mice without treatment (Figure 6A). Gene ontology (GO) term analysis demonstrated that, in addition to regulation of gene expression, inflammation-related features were enriched, including microglial activation involved in immune response, astrocyte activation, synapse pruning, and complement-mediated synapse pruning, as well as processes responsible for phagocytosis, engulfment, and Aβ clearance (Figure 6B). In agreement with previous reports (Heneka et al., 2014), deposition of Aβ in patients with AD is associated with innate immune system activation. Together, these data suggested that AP5 is of great importance to regulate inflammation in disease progression.

Since microglia and astrocyte are the main cell types exerting inflammatory effects in the brain, we carried out heatmaps of disease-associated microglia (DAM) genes from hippocampal RNA-seq data. We analyzed the gene expression profile in the hippocampus and observed an increase in DAM gene expression in APP/PS1 mice with AP5 treatment compared with APP/PS1 mice without treatment (Figure 6C), further suggesting that microglial cells acquired a typical DAM phenotype. The ability of microglia to switch to a DAM phenotype appears essential to limit Aβ plaque deposition and inflammation, thereby removing the neuronal damage in AD (Keren-Shaul et al., 2017). Moreover, CD11c (encoded by *Itgax*) has recently been identified as a marker for DAM (Keren-Shaul et al., 2017). We found that CD11c-positive microglia were accumulated around Aβ plaques. Notably, the CD11c-positive area within the activated microglia was significantly elevated with AP5 treatment (Figures 6D,E), further confirming that AP5 skewed microglia to a DAM phenotype and contributed to the decreased deposition of Aβ and inflammation.

At the same time, we compared the transcriptional profile of reactive astrocytes with that of astrocytes subpopulations recently identified in pan-reactive, A1 and A2, specific astrocytes (Liddelow et al., 2017). A1-specific astrocytes, induced by neuroinflammation, are powerfully neurotoxic, where their presence may well account for neurodegeneration and can drive disease deterioration. However, A2 astrocytes upregulate many neurotrophic factors and therefore are protective. As expected, astrocytes in the hippocampus of APP/PS1 mice displayed an increased gene expression of pan-reactive and A1-specific astrocytes signatures. AP5 reduced the expression of most genes linked to pan-reactive and A1 astrocytes (Figure 6F), suggesting that AP5 could prevent or revert A1-specific astrocytes formation. No differences among groups were detected in the expression of signature genes of A2-specific astrocytes. Since complement C3 (C3) is one of the most distinctive and highly elevated genes in A1-specific astrocytes and is not expressed by A2-specific astrocytes (Litvinchuk et al., 2018), we performed immunofluorescence to identify whether C3-expressing A1 astrocytes are decreased following AP5 treatment. We found that C3-positive area within the astrocytes was markedly reduced after AP5 treatment (Figures 6G,H), further confirming that AP5 could prevent and reverse A1-specific astrocyte formation. Taken together, our data demonstrated that the regulation of astrocyte and microglia phenotype transition by AP5 may be an important mechanism to control inflammation in AD.

## Discussion

While it is clear that the fundamental pathology of AD is driven by Aβ toxicity, neurofibrillary tangles, and neuroinflammation, recent clinical results suggest that addressing individual components of AD pathology may not be efficacious to treat the majority of AD (Long and Holtzman, 2019; Livingston et al., 2020). Therefore, the MTDLs aiming at several underlying causes of neurodegenerative disease in parallel presents an opportunity for successful AD treatment (Guzior et al., 2015; Savelieff et al., 2019). In our previous studies (Bai et al., 2019; Sang et al., 2019; Wang et al., 2019), a series of chalcone-O-carbamate derivatives and indanone-chalcone hybrid compounds were designed and synthesized as multifunctional agents for the treatment of AD. All these compounds were evaluated by AChE and BuChE inhibition, antioxidant activity, and Aβ aggregation inhibition *in vitro*. Among these synthesized derivatives, we found that target compounds with the *N*-ethyl-*N*-methylamine group (carbamate moieties) showed good eeAChE and eqBChE inhibitory activities in the carbamate moieties at the 3 position of the chalcone. Moreover, indanone–chalcone hybrid compounds exhibited the inhibitory potency of Aβ aggregation and disaggregation activity. To this end, we utilized the chalcone scaffold for the integration of carbamate moiety and dimethoxy-indanone moiety to generate a novel MTDL—AP5.

AP5 was able to span the active gorge of AChE and it exhibits a high affinity for CAS and PAS. This observation is supported by our studies that demonstrated its inhibition of AChE catalytic activity and AChE-induced Aβ aggregation *in vitro* and its ability to increase extracellular ACh concentration in mouse brains. Furthermore, AP5 displayed favorable drug-likeness properties and desirable PK characteristics including oral bioavailability (67.2%) and >10% brain penetrance.

AP5 could markedly inhibit AChE-induced Aβ aggregation *in vitro* and brain AChE catalytic activity in AD mice, which was consistent with the molecular docking result showing that AP5 could bind AChE through PAS and CAS. More importantly, AP5 was also capable of reducing Aβ deposition by influencing Aβ aggregation but not through APP processing in APP/PS1 mice. Yet, these findings may be contradictory to a prior report that increased levels of ACh could agonize M1 muscarinic acetylcholine receptor in the brain, and consequently result in the decrease of APP processing (Qiu et al., 2003). However, Nitsch and co-workers subsequently found chronic treatment with M1-selective agonists AF102B markedly lowered cerebrospinal fluid (CSF) Aβ levels in patients with AD, while physostigmine (an AChEI) did not show a significant effect on CSF Aβ in the same study as AF102B (Nitsch et al., 2000). This finding is consistent with a prior preclinical study (Farber et al., 1995), which showed that physostigmine did not change APP processing in rat brains. Moreover, the stimulation of M1 and M3 muscarinic receptors increases α-secretase APP processing, but simultaneous stimulation of M2 receptors blocks the M1 effect (Nitsch et al., 1992). In this context, although AP5 elevates Ach level, the effect is non-specific and extends to all muscarinic receptors. Thus, AP5 did not alter APP processing.

The compounds based on the inhibition of AChE cholinergic and non-cholinergic function, dual inhibitors of AChE, may exert dual therapeutic effects in symptom-relieving and disease-modifying manners (Viayna et al., 2021). The most potent dual AChEI reported so far is a dimer of indol and 6-chlorotacrine pharmacophore, named NP-61, that has a marked inhibition effect on AChE catalytic activity and great inhibition of AChE-induced Aβ fibrillization *in vitro*. NP-61 has been shown to display not only a cognitive improvement but also leads to a decrease in the plaque load in mice models (García-Palomero et al., 2008). Similar to the effect of NP-61, the present investigations strongly imply that AP5 targeting simultaneously the dual binding site of AChE offers an additive or synergistic therapeutic effect for AD. In an *in vivo* study, Thioflavin S staining and ELISA results confirmed that AP5 treatment significantly reduced brain fibrillar amyloid plaques load and insoluble Aβ aggregates in FA-fractions. Moreover, our results showed that AP5 can reduce the Aβ burden *via* eliciting microglia activation and recruiting microglia to Aβ deposition. Furthermore, studies *in vitro* revealed that AP5 significantly inhibited Aβ oligomerization or fibrillization. However, AP5 had no significant effects on the expression of APP, CTFs, BACE, and soluble Aβ in TBS-fraction. As illustrated above, we concluded that AP5 decreased Aβ plaque deposition by interfering with Aβ aggregation and promoting microglial Aβ phagocytosis without altering APP processing. Although it has proved that AP5 inhibited Aβ oligomerization or fibrillization *in vitro*, its interpretation that AP5 mitigated Aβ pathology by interfering with Aβ aggregation still has some limitations. Thus, in the future, confirming the inhibition and disaggregation effects of AP5 on Aβ aggregates in the AD mice model would strengthen our conclusions.

Neuroinflammation is a prevalent feature across neurodegenerative diseases and implicated in the progression of neurodegeneration (Leng and Edison, 2021). Hence, MTDLs simultaneously against AChE and neuroinflammation are expected to have more desired therapy potency (Li et al., 2009). To this end, we furthermore focused our study on dual-acting drugs that link AChE inhibitory and an anti-inflammatory response in a hybrid molecule. In our work, the inhibitory effect of AP5 on inflammatory effect is confirmed by reducing microglial and astrocyte activation-triggered morphological changes and the secretion of pro-inflammatory cytokines.

Microglia clustered around the Aβ plaque not only form a protective barrier but also promote the clearance of Aβ through phagocytosis and degradation (Yuan et al., 2016). Likewise, the suppression of pro-inflammatory cytokines secretion restores microglial phagocytosis toward Aβ in APP/PS1 the mice brain (Heneka et al., 2010). Just recently, there is a growing recognition that microglia constitute intercellular networks for enhanced clearance of intake pathological aggregation to control and alleviate microglial inflammatory effects and cytotoxicity (Scheiblich et al., 2021). In line with previous studies, our data demonstrated that AP5 enhanced microglia clustering around Aβ plaque, thereby diminishing plaque-associated neurotoxicity and promoting microglia Aβ phagocytosis and degradation in the brain of APP/PS1 mice. The transcription data further indicated a conversion of activated microglia toward a DAM phenotype following AP5 treatment. Hence, our results revealed that AP5 elicited morphological, functional, and transcriptional changes of microglia to endow them with enhanced capacity to clear Aβ aggregates and decrease neuroinflammation.

Reactive astrocytes are a cellular component of gliosis in neurodegenerative disorders. In AD, astrocytes could also ingest Aβ peptides to induce degradation pathways such as autophagy (Pomilio et al., 2016). However, reactive astrocytes are more involved in the neuron support and the modulation of neuronal signaling relative to its phagocytic activities or degrading functions (Santello et al., 2019). Thus, there was no marked change in the number of astrocytes around Aβ plaque. Moreover, AP5 could prevent or revert A1 astrocytes formation, which may ultimately lead to a more favorable neuronal milieu and improved neuronal and synaptic loss in the AD brain.

The absence of cognitive benefit in clinical trials targeting Aβ in the late stages of AD may account for the fact that neurodegeneration, neuronal death, and brain lesion(s) are too serious to be relieved (Golde et al., 2018). Hence, the most effective therapeutic strategy is likely to be prevention. To evaluate whether AP5 has the capacity to slow the memory deficits in AD mice models, we chronically administered AP5 to 6-month-old APP/PS1 mice, which were at the early stage of β-amyloidosis for 5 consecutive months. By a series of behavioral tests, we found AP5 exhibited the amelioration of cognitive decline. Overall, AP5 exerts multiple beneficial effects by targeting AChE and neuroinflammation from the early stage of AD.

## Conclusion

In conclusion, this study identifies a novel MTDL candidate AP5 and demonstrated that AP5 is a dual inhibitor of AChE to constrain the hydrolysis of ACh and Aβ plaque deposition in APP/PS1 mice model. In addition to AChE inhibitory activity, AP5 can suppress neuroinflammation by regulating microglia and astrocyte phenotype transition, which may account for the rescue of neuron and synapse loss, and also cognitive deficits. Together, our findings shed light on the therapeutic potency of AP5 for multifactorial disorders and provide a new solution for the treatment of AD.

## Data Availability Statement

The data presented in this study are deposited in online repository: https://pan.baidu.com/s/1pgjJSYvY6mQkFFQsmAILfQ?pwd=rjxt.

## Ethics Statement

The animal study was reviewed and approved by the Institutional Animal Care and Use Committees of Zunyi medical university.

## Author Contributions

CL performed the experiments, analyzed the data, and prepared the manuscript. YQ and JS provided intellectual support in experiment design, supervised the studies, and reviewed the manuscript. ZS designed and synthesized the novel MTDL drug. HP detected the novel MTDL concentration in plasma and brain homogenates by UPLC-TSQ/MS. QW provided valuable resource of instrument acquisition. All authors contributed to the article and approved the submitted version.

## Conflict of Interest

The authors declare that the research was conducted in the absence of any commercial or financial relationships that could be construed as a potential conflict of interest.

## Publisher’s Note

All claims expressed in this article are solely those of the authors and do not necessarily represent those of their affiliated organizations, or those of the publisher, the editors and the reviewers. Any product that may be evaluated in this article, or claim that may be made by its manufacturer, is not guaranteed or endorsed by the publisher.

## Footnotes

1
https://www.jax.org/strain/004462

## Abbreviations

AD: Alzheimer’s disease
MTDL: multi-target-directed ligand
AChE: acetylcholinesterase
ACh: acetylcholine
PAS: peripheral anionic site
CAS: catalytic anionic site
BBB: blood-brain barrier
A β: Amyloid- β
ThS: Thioflavin S
ThT: Thioflavin T
APP: amyloid precursor protein
CTFs: C-terminal fragments
BACE: β-site -APP cleaving enzyme
Iba1: ionized calcium-binding adapter molecule 1
GFAP: glial fibrillary acidic protein
DAM: disease-associated microglia
PK: pharmacokinetics
MWM: Morris water maze.

## References

[B1] Bai P. Wang K. Zhang P. Shi J. Cheng X. Zhang Q. (2019). Development of chalcone-O-alkylamine derivatives as multifunctional agents against Alzheimer’s disease. *Eur. J. Med. Chem.* 183:111737. 10.1016/j.ejmech.2019.111737 31581002

[B2] Benek O. Korabecny J. Soukup O. (2020). A perspective on multi-target drugs for Alzheimer’s Disease. *Trends Pharmacol. Sci.* 41 434–445. 32448557 10.1016/j.tips.2020.04.008

[B3] Castro A. Martinez A. (2001). Peripheral and dual binding site acetylcholinesterase inhibitors: implications in treatment of Alzheimer’s disease. *Mini. Rev. Med. Chem.* 1 267–272. 12369973 10.2174/1389557013406864

[B4] Chaudhaery S. S. Roy K. K. Shakya N. Saxena G. Sammi S. R. Nazir A. (2010). Novel carbamates as orally active acetylcholinesterase inhibitors found to improve scopolamine-induced cognition impairment: pharmacophore-based virtual screening, synthesis, and pharmacology. *J. Med. Chem.* 53 6490–6505. 10.1021/jm100573q 20684567

[B5] Chun H. Im H. Kang Y. J. Kim Y. Shin J. H. Won W. (2020). Severe reactive astrocytes precipitate pathological hallmarks of Alzheimer’s disease *via* H(2)O(2)(-) production. *Nat. Neurosci.* 23 1555–1566. 10.1038/s41593-020-00735-y 33199896

[B6] Collaborators G. B. D. D. (2019). Global, regional, and national burden of Alzheimer’s disease and other dementias, 1990-2016: a systematic analysis for the Global Burden of Disease Study 2016. *Lancet Neurol.* 18 88–106. 30497964 10.1016/S1474-4422(18)30403-4 PMC6291454

[B7] Farber S. A. Nitsch R. M. Schulz J. G. Wurtman R. J. (1995). Regulated secretion of beta-amyloid precursor protein in rat brain. *J. Neurosci.* 15 7442–7451. 7472496 10.1523/JNEUROSCI.15-11-07442.1995 PMC6578089

[B8] Ferreira S. T. Lourenco M. V. Oliveira M. M. De Felice F. G. (2015). Soluble amyloid-β oligomers as synaptotoxins leading to cognitive impairment in Alzheimer’s disease. *Front. Cell Neurosci.* 9:191. 10.3389/fncel.2015.00191 26074767 PMC4443025

[B9] Galdeano C. Viayna E. Arroyo P. Bidon-Chanal A. Blas J. R. Muñoz-Torrero D. (2010). Structural determinants of the multifunctional profile of dual binding site acetylcholinesterase inhibitors as anti-Alzheimer agents. *Curr. Pharm. Des.* 16 2818–2836. 10.2174/138161210793176536 20698824

[B10] García-Palomero E. Muñoz P. Usan P. Garcia P. Delgado E. De Austria C. (2008). Potent beta-amyloid modulators. *Neurodegener. Dis.* 5 153–156. 18322376 10.1159/000113688

[B11] Golde T. E. DeKosky S. T. Galasko D. (2018). Alzheimer’s disease: The right drug, the right time. *Science* 362 1250–1251. 30545877 10.1126/science.aau0437

[B12] Guo T. Zhang D. Zeng Y. Huang T. Y. Xu H. Zhao Y. (2020). Molecular and cellular mechanisms underlying the pathogenesis of Alzheimer’s disease. *Mol. Neurodegener.* 15:40. 10.1186/s13024-020-00391-7 PMC7364557 32677986

[B13] Gupta M. Lee H. J. Barden C. J. Weaver D. F. (2019). The Blood-Brain Barrier (BBB) Score. *J. Med. Chem.* 62 9824–9836. 31603678 10.1021/acs.jmedchem.9b01220

[B14] Guzior N. Wieckowska A. Panek D. Malawska B. (2015). Recent development of multifunctional agents as potential drug candidates for the treatment of Alzheimer’s disease. *Curr. Med. Chem.* 22 373–404. 25386820 10.2174/0929867321666141106122628 PMC4435057

[B15] Heneka M. T. Kummer M. P. Latz E. (2014). Innate immune activation in neurodegenerative disease. *Nat. Rev. Immunol.* 14 463–477. 24962261 10.1038/nri3705

[B16] Heneka M. T. Nadrigny F. Regen T. Martinez-Hernandez A. Dumitrescu-Ozimek L. Terwel D. (2010). Locus ceruleus controls Alzheimer’s disease pathology by modulating microglial functions through norepinephrine. *Proc. Natl. Acad. Sci. U.S.A.* 107 6058–6063. 10.1073/pnas.0909586107 20231476 PMC2851853

[B17] Inestrosa N. C. Alvarez A. Pérez C. A. Moreno R. D. Vicente M. Linker C. (1996). Acetylcholinesterase accelerates assembly of amyloid-beta-peptides into Alzheimer’s fibrils: possible role of the peripheral site of the enzyme. *Neuron* 16 881–891. 10.1016/s0896-6273(00)80108-7 8608006

[B18] Jan A. Hartley D. M. Lashuel H. A. (2010). Preparation and characterization of toxic Abeta aggregates for structural and functional studies in Alzheimer’s disease research. *Nat. Protoc.* 5 1186–1209. 20539293 10.1038/nprot.2010.72

[B19] Johnson G. Moore S. W. (2006). The peripheral anionic site of acetylcholinesterase: structure, functions and potential role in rational drug design. *Curr. Pharm. Des.* 12 217–225. 10.2174/138161206775193127 16454738

[B20] Kayed R. Head E. Thompson J. L. McIntire T. M. Milton S. C. Cotman C. W. (2003). Common structure of soluble amyloid oligomers implies common mechanism of pathogenesis. *Science* 300 486–489. 12702875 10.1126/science.1079469

[B21] Keren-Shaul H. Spinrad A. Weiner A. Matcovitch-Natan O. Dvir-Szternfeld R. Ulland T. K. (2017). A unique microglia type associated with restricting development of Alzheimer’s Disease. *Cell* 169 1276–1290.e17. 10.1016/j.cell.2017.05.018 28602351

[B22] Leng F. Edison P. (2021). Neuroinflammation and microglial activation in Alzheimer disease: where do we go from here? *Nat. Rev. Neurol.* 17 157–172. 10.1038/s41582-020-00435-y 33318676

[B23] Li J. Hu J. Shao B. Zhou W. Cui Y. Dong C. (2009). Protection of PMS777, a new AChE inhibitor with PAF antagonism, against amyloid-beta-induced neuronal apoptosis and neuroinflammation. *Cell Mol. Neurobiol.* 29 589–595. 10.1007/s10571-009-9351-0 19194797 PMC11506095

[B24] Liddelow S. A. Guttenplan K. A. Clarke L. E. Bennett F. C. Bohlen C. J. Schirmer L. (2017). Neurotoxic reactive astrocytes are induced by activated microglia. *Nature* 541 481–487. 28099414 10.1038/nature21029 PMC5404890

[B25] Lipinski C. A. Lombardo F. Dominy B. W. Feeney P. J. (2001). Experimental and computational approaches to estimate solubility and permeability in drug discovery and development settings. *Adv. Drug Deliv. Rev.* 46 3–26. 10.1016/s0169-409x(00)00129-0 11259830

[B26] Litvinchuk A. Wan Y. W. Swartzlander D. B. Chen F. Cole A. Propson N. E. (2018). Complement C3aR inactivation attenuates tau pathology and reverses an immune network deregulated in tauopathy models and Alzheimer’s Disease. *Neuron* 100 1337–1353.e5. 10.1016/j.neuron.2018.10.031 30415998 PMC6309202

[B27] Livingston G. Huntley J. Sommerlad A. Ames D. Ballard C. Banerjee S. (2020). Dementia prevention, intervention, and care: 2020 report of the Lancet Commission. *Lancet* 396 413–446. 32738937 10.1016/S0140-6736(20)30367-6 PMC7392084

[B28] Long J. M. Holtzman D. M. (2019). Alzheimer Disease: An Update on Pathobiology and Treatment Strategies. *Cell* 179 312–339. 31564456 10.1016/j.cell.2019.09.001 PMC6778042

[B29] Mezeiova E. Chalupova K. Nepovimova E. Gorecki L. Prchal L. Malinak D. (2019). Donepezil Derivatives Targeting Amyloid-β Cascade in Alzheimer’s Disease. *Curr. Alzheimer Res.* 16 772–800. 10.2174/1567205016666190228122956 30819078

[B30] Mondal P. Gupta V. Das G. Pradhan K. Khan J. Gharai P. K. (2018). Peptide-based acetylcholinesterase inhibitor crosses the blood-brain barrier and promotes neuroprotection. *ACS Chem. Neurosci.* 9 2838–2848. 10.1021/acschemneuro.8b00253 30015476

[B31] Nitsch R. M. Deng M. Tennis M. Schoenfeld D. Growdon J. H. (2000). The selective muscarinic M1 agonist AF102B decreases levels of total Abeta in cerebrospinal fluid of patients with Alzheimer’s disease. *Ann. Neurol.* 48 913–918. 11117548

[B32] Nitsch R. M. Slack B. E. Wurtman R. J. Growdon J. H. (1992). Release of Alzheimer amyloid precursor derivatives stimulated by activation of muscarinic acetylcholine receptors. *Science* 258 304–307. 1411529 10.1126/science.1411529

[B33] Pérez D. I. Martínez A. Gil C. Campillo N. E. (2015). From bitopic inhibitors to multitarget drugs for the future treatment of Alzheimer’s Disease. *Curr. Med. Chem.* 22 3789–3806. 10.2174/0929867322666150812145825 26264921

[B34] Pomilio C. Pavia P. Gorojod R. M. Vinuesa A. Alaimo A. Galvan V. (2016). Glial alterations from early to late stages in a model of Alzheimer’s disease: Evidence of autophagy involvement in Aβ internalization. *Hippocampus* 26 194–210. 10.1002/hipo.22503 26235241 PMC5467976

[B35] Qiu Y. Wu X. J. Chen H. Z. (2003). Simultaneous changes in secretory amyloid precursor protein and beta-amyloid peptide release from rat hippocampus by activation of muscarinic receptors. *Neurosci. Lett.* 352 41–44. 10.1016/j.neulet.2003.08.022 14615045

[B36] Sang Z. Wang K. Shi J. Liu W. Tan Z. (2019). Design, synthesis, in-silico and biological evaluation of novel chalcone-O-carbamate derivatives as multifunctional agents for the treatment of Alzheimer’s disease. *Eur. J. Med. Chem.* 178 726–739. 10.1016/j.ejmech.2019.06.026 31229875

[B37] Santello M. Toni N. Volterra A. (2019). Astrocyte function from information processing to cognition and cognitive impairment. *Nat. Neurosci.* 22 154–166. 30664773 10.1038/s41593-018-0325-8

[B38] Savelieff M. G. Nam G. Kang J. Lee H. J. Lee M. Lim M. H. (2019). Development of multifunctional molecules as potential therapeutic candidates for alzheimer’s disease. parkinson’s disease, and amyotrophic lateral sclerosis in the last decade. *Chem. Rev.* 119 1221–1322. 10.1021/acs.chemrev.8b00138 30095897

[B39] Scheiblich H. Dansokho C. Mercan D. Schmidt S. V. Bousset L. Wischhof L. (2021). Microglia jointly degrade fibrillar alpha-synuclein cargo by distribution through tunneling nanotubes. *Cell* 184 5089–5106.e21. 10.1016/j.cell.2021.09.007 34555357 PMC8527836

[B40] Schneider L. S. Geffen Y. Rabinowitz J. Thomas R. G. Schmidt R. Ropele S. (2019). Low-dose ladostigil for mild cognitive impairment: A phase 2 placebo-controlled clinical trial. *Neurology* 93 e1474–e1484. 10.1212/WNL.0000000000008239 31492718 PMC7010322

[B41] Shi Y. Huang W. Wang Y. Zhang R. Hou L. Xu J. (2018). Bis(9)-(-)-Meptazinol, a novel dual-binding AChE inhibitor, rescues cognitive deficits and pathological changes in APP/PS1 transgenic mice. *Transl Neurodegener* 7 21. 10.1186/s40035-018-0126-8 30237878 PMC6142624

[B42] Viayna E. Coquelle N. Cieslikiewicz-Bouet M. Cisternas P. Oliva C. A. Sánchez-López E. (2021). Discovery of a Potent Dual Inhibitor of Acetylcholinesterase and Butyrylcholinesterase with Antioxidant Activity that Alleviates Alzheimer-like Pathology in Old APP/PS1 Mice. *J. Med. Chem.* 64 812–839. 10.1021/acs.jmedchem.0c01775 33356266

[B43] Wang K. Yu L. Shi J. Liu W. Sang Z. (2019). Multifunctional indanone–chalcone hybrid compounds with anti-β-amyloid (Aβ) aggregation, monoamine oxidase B (MAO-B) inhibition and neuroprotective properties against Alzheimer’s disease. *Med. Chem. Res.* 28 1912–1922.

[B44] Young A. L. Marinescu R. V. Oxtoby N. P. Bocchetta M. Yong K. Firth N. C. (2018). Uncovering the heterogeneity and temporal complexity of neurodegenerative diseases with Subtype and Stage Inference. *Nat. Commun.* 9:4273. 10.1038/s41467-018-05892-0 30323170 PMC6189176

[B45] Yuan P. Condello C. Keene C. D. Wang Y. Bird T. D. Paul S. M. (2016). TREM2 haplodeficiency in mice and humans impairs the microglia barrier function leading to decreased amyloid compaction and severe axonal dystrophy. *Neuron* 92 252–264. 27710785 10.1016/j.neuron.2016.09.016

[B46] Zhong L. Xu Y. Zhuo R. Wang T. Wang K. Huang R. (2019). Soluble TREM2 ameliorates pathological phenotypes by modulating microglial functions in an Alzheimer’s disease model. *Nat. Commun.* 10:1365. 10.1038/s41467-019-09118-9 PMC6433910 30911003

[B47] Zhuang C. Zhang W. Sheng C. Zhang W. Xing C. Miao Z. (2017). Chalcone: a privileged structure in medicinal chemistry. *Chem. Rev.* 117 7762–7810. 28488435 10.1021/acs.chemrev.7b00020 PMC6131713

