# Supplementary material for: A Novel Multifunctional 5,6-Dimethoxy-Indanone-Chalcone-Carbamate Hybrids Alleviates Cognitive Decline in Alzheimer’s Disease by Dual Inhibition of Acetylcholinesterase and Inflammation
Source: Front Aging Neurosci. 2022 Jul 4;14:922650. doi: 10.3389/fnagi.2022.922650 (PMC9289467; doi:10.3389/fnagi.2022.922650)
Supplement: Supplementary file 1 [file Presentation_1.pdf]

Supplementary Material

Table of contents

Supplementary materials and methods……………………………………………...1

Supplementary table 1……………….………………………………………...6

Supplementary table 2……………….………………………………………...6

Supplementary Figure 1…………………………………………………….....7

Supplementary Figure 2…………………………………………………….....7

Supplementary materials and methods

Prepations of specimen for pharmacokinetics analysis

Whole-blood samples were collected at 0, 0.083, 0.25, 0.5, 1, 1.5, 2, 3, 4, 7, 8, 16 and 24 h after dosing. Brains were collected at 0, 0.25, 0.5, 2, 4, 8 and 24 h after administration. Plasma extractions were performed by adding 90 μL methanol and 2 μL internal standard letrozole (500 ng/mL) to a 30 μL plasma sample. Brain samples were homogenized in 1 mL PBS per gram brain tissue by handheld homogenizer. Then 90 μL methanol and 2 μL letrozolewere added to 30 μl brain homogenates. The samples were vortexed for 3 min and centrifuged 15 min at 25,000 g. The plasma and brain homogenate were analyzed by UPLC-TSQ/MS (Thermo Fisher Scientific, Waltham, MA, USA). Samples were compared to a calibration curve prepared in a similar manner by spiking blank control plasma or homogenate with standards prepared in methanol and then extracted as above.

Amyloid-β fibril aggregation and disaggregation determination by Thioflavin T assay

Synthetic human Aβ40 (03-136, Invitrogen, Waltham, MA) or Aβ42 (A118755, Aladdin, Shanghai, China) peptides were dissolved in hexafluoro-2-iso-propanol (HFIP, H107503, Aladdin) to make a solution of 1 mg/mL. HFIP was removed by

evaporation to form film. Then Aβ film was dissolved in anhydrous DMSO to make a 100 μM fibril solution.

To study the kinetics of Aβ-AChE fibril complex formation, 10 μM Aβ was mixed with AChE (R005282, Roche, Basel, Switzerland) and different concentrations AP5 and incubated with 10 μM ThT and 50 mM glycine-NaOH (pH 8.5) at 37°C, the ThT fluorescence was continually measured for 24 h at 30 min intervals. For the disaggregation of pre-formed Aβ-AChE fibril complex, the Aβ and AChE (Aβ final concentration 10 μM, Aβ/AChE molar ratio 100:1) solution was incubated at 37°C for 48 h. AP5 at various concentrations was then added to the pre-aggregated Aβ solution and incubated for another 48 h at 37°C. In ThT-binding measurements, mix 80 μl of Aβ-AChE plus AP5 fraction with 10 μl of 100 μM ThT and 10 μl of 500 mM glycine-NaOH. Fluorescence emission at 485 nm was measured on a Spectra MAX GeminiXS fluorometerat an excitation wavelength of 450 nm. For each determination, the fluorometer was calibrated by ThT reagent alone.

Behavioral tset

Open field test

To assess locomotor activity, mice were individually tested in an open-field box (38 cm × 38 cm × 25 cm). The movement of the mouse was tracked for 5 min with open field video analysis system, and the total move distances was automatically recorded as a direct measure of locomotor activity. Between each trial, the walls and floor were wiped with 75% ethanol.

Novel object recognition, spatial object location

For the novel object recognition task, mice were individually exposed to the configuration of two identical objects (A+A) in open field during a 5 min acquisition trial. Exactly 1 h or 24 h later, during the retention sessions, mice were placed back into the same arena where they were exposed to one familiar object A and to a novel object B (after 1 h) and object C (after 24 h). The chamber was cleaned with 75% ethyl alcohol to eliminate odor before the next test. The discrimination index was calculated and defined as: (time spent exploring the new object-time spent exploring

the familiar object) / total exploration time for the novel and familiar object.

For the spatial object location task (an object moved to a new location), mice first explored the two objects placed in two corners of the open field for 5 min in training period. After 1 h or 24 h, the mice received a 5 min retention trial with a new spatial configuration with an object moved from one corner to the opposite corner of the open field. The discrimination index was calculated and defined as: (time spent exploring the displaced object-time spent exploring nondisplaced objects) / total exploration time for the two objects during the 5 min retention trial.

Y-maze

Work on spatial recognition memory was tested by Y-maze. The symmetrical Y-maze consisted of three arms joined in the middle to form a Y shape. During the training trial, one of the arms (novel arm) was closed, and each mouse was placed in the start arm and allowed to explore the start arm freely for 5 min. One hour later, the testing trial was performed, and all three arms were open. Mice were allowed to explore the three arms for 5 min. The ratio between the distance spent in the novel arm and the total distance travelled in the maze was quantified as preference index.

Morris water maze (MWM)

The effect of AP5 on spatial learning and memory performance of mice was tested by the MWM. Briefly, during the training period, the mice were allowed to freely swim for 60 s to search for the platform (10 cm in diameter, 2 cm beneath the water surface), which was fixed in the center of one of the four quadrants. Mice went through three trials each day and were alternated among three random quadrants for five consecutive days. Mice that failed to find the platform within 60 s were manually guided to platform and allowed to stay on it for 30 s. The time (escape latency) for each mouse to find the way and reach the target platform was analyzed. For the probe trial, 24 h after the last training session, the platform was removed and the mice were allowed to swim for 60 s. The parameters measured during the probe trial included number of platform crossing and time spent in the target quadrant.

Estimation of brain AChE and acetylcholine (ACh)

Briefly, for ACh assay, the reactions were started by adding 100 μL of the Amplex Red reagent, HRP, choline oxidase, AChE working solution to each microplate well containing the controls and samples. For AChE assay, detection cocktail solution containing ACh, Amplex Red, choline oxidase and horseradish peroxidase was added to each well. Assay plates were incubated at room temperature for 30 min and protected from light, followed by detecting fluorescence of resorufin (λex = 544 nm, λem = 590 nm) in a fluorescence microplate reader.

Immunohistochemistry

After deparaffinization and antigen retrieval, the sections were incubated with 0.3% hydrogen peroxide (H_2O_2) to inhibit endogenous peroxidase activity for 20 min and blocked by goat serum for 30 min at 37°C. Slides were subsequently incubated overnight with specific primary antibodies: anti-Iba1(1:100, ab178847, Abcam, Cambridge, UK) or anti-GFAP (1:100, bs-0199R, Bioss) at 4 °C. After being washed with PBS, slides were treated with a biotinylated secondary antibody for 30 min and with streptavidin-peroxidase complex for another 30 min at 37°C. Immunostaining was visualized with 3,3′-diaminobenzidine (DAB, ZL1-9018, Zhongshan Jinqiao, Beijing, China). The images were acquired using a Nikon microscope (Nikon, Melville, NY, USA).

Immunofluorescence

After deparaffinization and antigen retrieval, slides were incubated in a blocking solution consisting of 10% normal goat serum and 0.4% Triton diluted in PBS for 1 hour at room temperature, followed by primary antibodies overnight at 4 °C. Primary antibodies used in this study include anti-β amyloid (MOAB-2, NBP2-13075SS, Novus Biologicals), anti-Iba1(1:100, ab178847, Abcam), anti-GFAP (1:100, bs-0199R, Bioss), anti-CD68 (1:50, bs0649R, Bioss), anti-MAP2 (1:100, 17490-1-AP, Proteintech, WuHan, China), anti-NeuN (1:100, 26975-1-AP, Proteintech), anti-postsynaptic density95 (PSD95, 1:50, 20665-1-AP, Proteintech) and anti-synaptophysin (1:50, 67864-1-lg, Proteintech), anti-CD11c (1:100, 97585, Cell

Signaling Technology, Danvers, MA, USA), anti-Complement Component C3d (1:100, BAF2655, R&D, Minneapolis, MN, USA). The slides were rinsed three times in PBS. Then the slides were labeled with fluorescent secondary antibodies as follows: goat anti-rabbit IgG H&L Alexa Fluor® 488 (ab150077, Abcam), goat anti-rabbit IgG H&L Alexa Fluor® 594 (ab150080, Abcam) or goat anti-mouce IgG H&L Alexa Fluor® 594 (ab150116, Abcam). Slides were washed three times with PBS before being mounted with DAPI fluoromount. Images were captured using an Olympus SpinSR10 scanner. For the quantification of Aβ plaque-associated microglia and astrocyte, Iba1- and GFAP-immunopositive cells around plaques were counted. On each slide, ten plaques were randomly determined and the microglia (nuclei) in the immediate vicinity (two times the plaque diameter) of each plaque were counted. For colocalization of PSD95 and synaptophysin, images were analyzed with the puncta analyzer plugin with a minimum pixel specification of 4 in Image J and thresholding was consistent for every image.

Western blotting

Protein samples was separated in 10% or 12% SDS-PAGE and then transferred to PVDF membranes. The membranes were incubated with 5% non-fat milk blocking solution for 1 h at room temperature, and then probed with primary antibodies overnight at 4°C, including anti-amyloid precursor protein full length (APPfl, ab180140, Abcam), anti-BACE (1:1000, sc-33711, Santa-Cruz Biotechnology, Dallas, TX), anti-CTF (1:1000, A8717, Sigma Aldrich), anti-presenilin1 (1;1000, ab76083, Abcam), anti-MAP2 (1:1000, 17490-1-AP, Proteintech), anti-NeuN (1:1000, 26975-1-AP, Proteintech), anti-PSD95 (1:1000, 20665-1-AP, Proteintech) and anti-synaptophysin (1:1000, 67864-1-lg, Proteintech) overnight at 4°C. Then the membranes were incubated with the corresponding HRP-conjugated secondary antibodies at room temperature for 1 h, and developed with ECL. Densitometric analysis was performed using Quantity One software (Bio-Rad, Berkeley, CA, USA). These analyses were normalized to β-tubulin (1:1000, 10494-1-AP, Proteintech).

Supplementary Table1

Predicted physicochemical values for oral absorption and blood-brain barrier penetration with Lipinski's rule of five and BBB score *in silico*

Lipinski's Rule of Five	Molecular weight	Log P	Hydrogen-bond Acceptor	Hydrogen-bond Donor	TPSA	BBB score (Threshold 0.02)
AP5	381.43	3.977	5	0	65.07	0.038
Rivastigmine	250.42	2.76	3	0	32.78	0.042
Donepezil	379.5	4.361	4	0	38.77	0.135

Generally, small molecules as desirable physicochemical properties specifically for CNS drugs may be considered to possess drug-likeness and potential BBB permeability if they have (i) ≤ 450Da molecular weight; (ii) logarithm of octanol−water partition coefficient (log P) < 4.0; (iii) ≤ 7 hydrogen bond acceptors; (iv) ≤ 2 hydrogen bond donors; (v) Topological surface area (TPSA) ≤ 70 Å2; (vi) BBB score>0.02.

Supplementary Table 2

Summary of single dose pharmacokinetic parameters of AP5 in mouse

Distribution	Dose Route	Dose (mg/kg)	C_{max} (ng/mL)	T_{max} (hour)	$t_{1/2}$ (hour)	AUC_{last} (hr•ng/mL)	AUC_{INF} (hr•ng/mL)	Cl (L/hr/kg)	V_{ss} (L/kg)	F (%)	B/P (%)
Plasma	*p.o.*	40	2088	2.0	4.725	6988	7257	2.756	18.79	67.2	
	i.v.	10	5443	0.083	3.341	2597	2698	7.524	36.27		
Brain	*p.o.*	40	95.39	1.5	5. 318	706.2	743.9	53.77	412.6		10.23

Data were subjected to non-compartmental analysis using DAS (Drug and Statistics) Version 3.0 . The following parameters and constants were determined: maximum plasma concentration (C_{max}), time to maximum plasma concentration (T_{max}), terminal phase elimination half-life ($t_{1/2}$), area under the plasma concentration-time curve to the last time point (AUC_{last}), area under the plasma concentration-time curve extrapolated to infinity (AUC_{INF}), volume of distribution at steady state (V_{ss}),and clearance (Cl). The extent of brain penetration were measured by brain-to-plasma distribution ratio (B/P).The oral B/P value was calculated as: $AUC_{INF\ brain}/AUC_{INF\ plasma} \times 100$. Bioavailability (F%) after oral administration was calculated as: $AUC_{INF\ P.O.}/AUC_{INF\ i.v.}\ /4 \times 100$.

Supplementary Figure 1

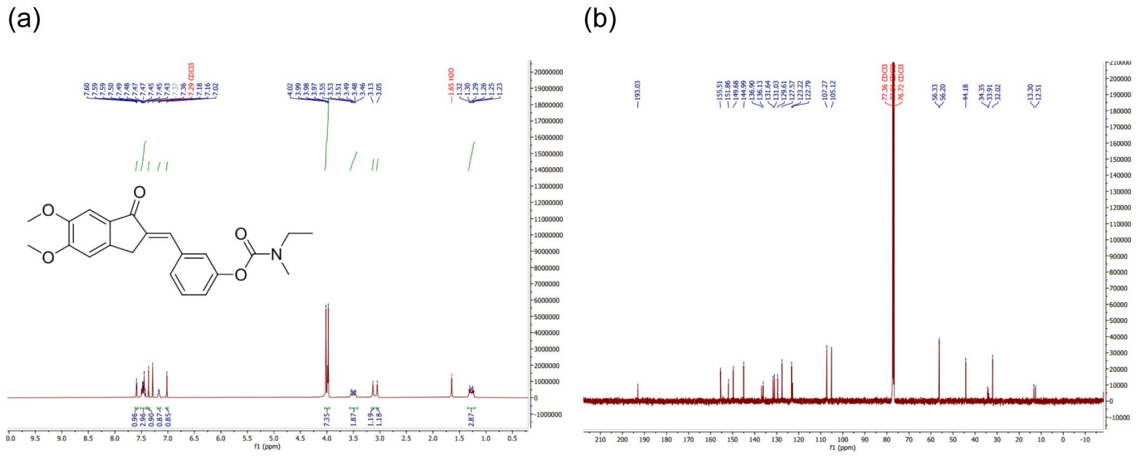

Supplementary Fig. 1 Mass spectra of *(E)*-3-((5,6-dimethoxy-1-oxo-1,3-dihydro-2H-Inden -2-ylidene)-methyl)phenylethyl(methyl)carbamate (named AP5). **(a)** ^{1}H NMR (400MHz) spectra of AP5 in Chloroform-*d*. **(b)** ^{13}C NMR (101MHz) spectra of AP5 in CDCl3.

Supplementary Figure 2

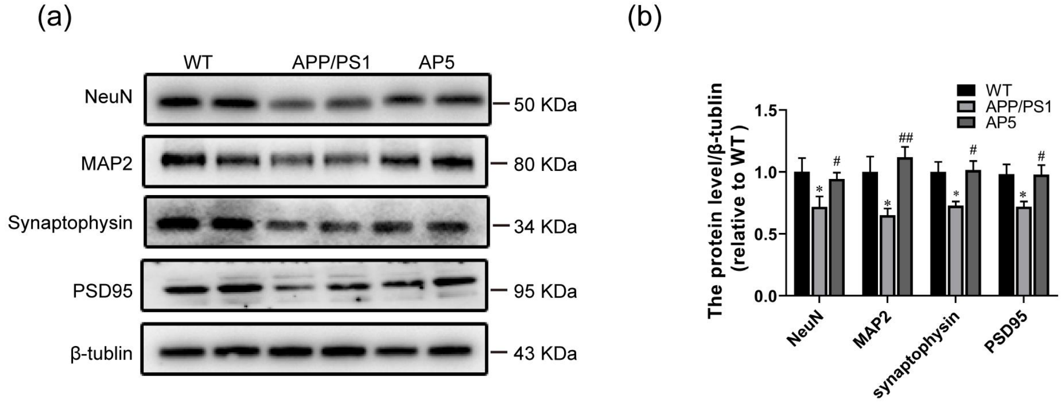

Supplementary Figure2 AP5 treatment increases neuronal and synaptic protein expresson in hippocampus of APP/PS1 mice. (**a)** Protein expression levels of NeuN, MAP2, synaptophysin and PSD95 in hippocampus were examined by Western blot .(**b)** Quantification of NeuN, MAP2, synaptophysin and PSD95 in hippocampus with Quantity One software. *P < 0.05 compared with WT mice, $^{\#}P$ < 0.05, $^{\#\#}P$ < 0.01 compared with APP/PS1 group by one-way ANOVA followed by a Turkey's *post-hoc* test, n = 6 mice per group.
